# Efficacy comparison of different moxibustion treatments for allergic rhinitis: A systematic review and Bayesian network meta-analysis

**DOI:** 10.1097/MD.0000000000032997

**Published:** 2023-03-03

**Authors:** Ke Chen, Chaofeng Hou, Chengjiang Liu, Yong Meng

**Affiliations:** a Second Clinical Medical College of Henan University of Chinese Medicine, Zhengzhou, China; b Department of general practice, Anhui Medical University, He Fei, China; c Henan Provincial Hospital of Traditional Chinese Medicine, Zhengzhou, China.

**Keywords:** allergic rhinitis, complementary and alternative therapies, meta-analysis, moxibustion, systematic review

## Abstract

At present, there are many commonly used moxibustion methods in clinical practice, but it is unclear which moxibustion is more suitable for the treatment of allergic rhinitis (AR), so we used a network meta-analysis to analyze the effectiveness of various moxibustion types in the treatment of AR.

**Methods:**

We searched 8 databases for comprehensive inclusion of randomized controlled trials (RCTs) for moxibustion in the treatment of allergic rhinitis. The search time was from the beginning of database establishment to January 2022. The Cochrane Risk of Bias tool was used to analyze the risk of bias of the included RCTs. Bayesian network meta-analysis of the included RCT was performed using the R software GEMTC and the RJAGS package.

**Results:**

A total of 38 RCTs were included, involving 4257 patients and 9 types of moxibustion. The network meta-analysis results revealed that heat-sensitive moxibustion (HSM) not only has the best effect in terms of effective rate (Odd ratio [OR]: 32.77, 95% Credible intervals [CrIs]: 1.86–1360.2) among the nine types of moxibustion, but also has good effect in improving the quality of life score (standard mean differences [SMD]: 0.6, 95% CrIs: 0.07–1.29). In terms of improving IgE and VAS scores, various types of moxibustion were comparable to the efficacy of western medicine.

**Conclusion:**

The results showed that HSM was the most effective treatment for AR compared with other types of moxibustion. Therefore, it can be regarded as a complementary and alternative therapy for AR patients with poor effects of traditional treatment and patients who are susceptible to adverse reactions of western medicine.

## 1. Introduction

Allergic rhinitis (AR) is a common allergic disease, and its main symptoms include pruritus, sneezing, nasal congestion and runny nose, which reduce the quality of life, sleep quality, work and learning efficiency of patients.^[1–5]^ AR affects 10% to 20% of the global population and the prevalence rate is increasing year by year.^[6]^ At the same time, AR will also cause a huge economic burden. According to statistics, the annual medical expenditure for the treatment of AR in the United States is $2–5 billion.^[7,8]^ In addition, AR indirectly affects productivity, according to a social study, symptoms and depression of AR patients are one of the main causes for absence from work in the spring.^[9]^

Currently, the commonly used first-line therapies for AR include glucocorticoids, oral or nasal antihistamines, and oral leukotriene receptor antagonists.^[10,11]^ Such drugs are prone to relapse and cannot change the process of allergic lesions after withdrawal, and also bring side effects such as sleepiness, dry mouth, dry eyes, constipation, and cardiotoxicity.^[12,13]^ Subcutaneous immunotherapy can change the development process of AR by regulating the immune system, but this therapy generally requires continuous treatment for 2 to 3 years, with slow onset and high price.^[7]^ Therefore, more and more people pay attention to complementary and alternative therapy.

Moxibustion treatment is an important part of Chinese Acupuncture and Moxibustion. This therapy places the ignited moxa wool or moxa pillar stick at the acupoint or the diseased region to prevent and treat the disease through its warm stimulation and drug effect. Moxibustion can dredge the channel, improve the fluid circulation, regulate the functions of viscera and improve immunity.^[14]^ Modern studies have proved that moxibustion can achieve anti-autoimmunity and anti-allergy functions by regulating the balance of the immune system in the body.^[15]^ Studies have shown that more and more AR patients choose acupuncture treatment.^[16]^

In the past 5 years, a number of systematic reviews have shown that different moxibustion treatment have effectively improved AR symptoms and improved the quality of life of patients.^[17–19]^ In 2021, Yin^[20]^ conducted a systematic evaluation and network meta-analysis on the treatment of allergic rhinitis by acupuncture, but the 9 intervention methods in this study only included 2 moxibustion treatments. However, there are many kinds of moxibustion used in the treatment of AR, including indirect moxibustion, governor meridian moxibustion, heat-sensitive moxibustion, warm moxibustion, thunder fire moxibustion and separate moxibustion. So far, there is no study comparing the efficacy and safety of various moxibustions in the treatment of AR. Therefore, the purpose of this study was to indirectly or directly compare the efficacy of different moxibustion treatments for AR through network meta-analysis and rank them according to the efficacy indicators, so as to provide a reference for clinical selection of moxibustion treatment schemes from the perspective of evidence-based medicine.

## 2. Information and methods

This study was prepared under the guidance of the Preferred Reporting Items for Systematic Evaluation and Meta-Analysis (PRISMA) guidelines^[21]^ (Supplementary file 1, Supplemental Digital Content, http://links.lww.com/MD/I500) In addition, the protocol of this study was registered on PROSPERO (ID: CRD42022311991).

### 2.1. Search strategies

Our search of computer databases included PubMed, Cochrane Library, Web of Science, Embase, CNKI, WanFang Data, VIP and CBMdisc. The retrieval period was from the beginning of repository construction until January 2022. In this study, subject words, free words and Boolean logic operator connection were used for retrieval without language restriction. The following keywords and MeSH terms were used for the search: “rhinitis,” “allergic,” “allergic rhinitis,” “rhinitis allergic” “moxibustion,” “moxabustion,” “moxa,” “mugwort.” The search strategy using PubMed database as an example is included in the Supplementary File Table S1, Supplemental Digital Content, http://links.lww.com/MD/I501.

### 2.2. Inclusion criteria

Randomized controlled trials (RCTs) of moxibustion in the treatment of allergic rhinitis, with no publication year and no language restriction. Participants: Patients with allergic rhinitis who met accepted diagnostic criteria, such as Guidelines for diagnosis and Treatment of Allergic rhinitis (2015, Tianjin)^[22]^ or Allergic Rhinitis and its Impact on Asthma (ARIA) 2008^[23]^ or Chinese society of allergy guidelines for the diagnosis and treatment of allergic rhinitis.^[24]^ Aged over 18 years old. Without race, sex, course of disease or region restriction. Interventions and Comparisons: The intervention methods of observation group were moxibustion related therapy, or moxibustion combined with other traditional Chinese medicine therapies. The intervention methods of the control group included Placebo or blank control, Conventional western medicine treatment, or the comparison between different moxibustion intervention measures. Definition table of different moxibustion therapies is in the Supplementary File Table S2, Supplemental Digital Content, http://links.lww.com/MD/I502. Outcomes: Primary outcome: the clinical effective rate. Secondary outcomes: rhino-conjunctivitis quality of questionnaire (RQLQ) scores,^[25,26]^ the level of immunoglobulin E (IgE), visual analog scale (VAS) scores.^[27]^

### 2.3. Exclusion criteria

1. Less than 18 years old or older than 65 years old; Combined Bronchial asthma, chronic obstructive pulmonary disease, bronchiectasis, rhinosinusitis, acute rhinitis, nasopharyngeal tumors, deviated septum, respiratory sensitivities sinusitis, acute rhinitis, nasopharyngeal tumor, deviated nasal septum, respiratory tract infection Infection; Duplicate publication; Psychiatric patients.
2. In the control group, the interventions were traditional Chinese medicine treatments such as acupuncture, manipulation, and Chinese herbal medicine, except for moxibustion.
3. Studies where the required data cannot be obtained.
4. RCT to study the acupuncture points, dosage and frequency of moxibustion.

### 2.4. Study screening and data extraction

After the included studies were imported into Endnote X9 software for automatic re-checking, two researchers independently screened the studies and then cross-checked them. Two researchers deleted irrelevant studies after reading titles and abstracts independently. Then they read the full text to screen the studies that met the requirements. If two researchers had different opinions in the screening process, a third party was invited to assist in judgment. Excel 2019 was used to create a spreadsheet to collect the extracted data. Data extraction was performed independently by two researchers. It included basic information about the included studies first author, year of publication, baseline information about the study population, interventions in the observation and control groups, duration of treatment, outcomes data, adverse events, number of dropouts and factors to evaluate risk of bias. Cross-checking between the two researchers after all the data has been extracted and third- party adjudication in case of disagreement.

### 2.5. Quality assessment

Two investigators used the Cochrane Collaborative Risk of Bias Tool to assess the quality of included studies.^[28]^ The quality assessment included the following seven main entries: generation of the randomization order; allocation concealment of the randomization scheme; blinding of study subjects and intervention implementers; blinding of outcome measures; completeness of outcome data; reporting selectivity bias; and other biases. For each of the seven items above, the included studies were classified as low risk, high risk or unclear. Two researchers completed the evaluation independently, and disputes were discussed with a third party to reach consensus.

### 2.6. Statistical analysis

R V.3.6.1 software and Stata version 15.1 were used to analyze the data and prepare the graphs. We used the R software GEMTC and RJAGS packages for Bayesian network meta-analysis. Considering the expected heterogeneity of the included studies, a random-effects model was used for statistical analysis. For dichotomous variables, the dominance ratio (OR) and 95% confidence interval (CI) were used to assess the effect size. Besides, effect sizes for continuous variables were assessed using standard mean differences (SMD) and 95% CIs. The effect size measure for the continuous outcome was chosen for SMD because the study used a different rating scale and unit of measure. The transferability hypothesis was assessed by comparing the distribution of potential effects study modifiers (year of publication, sample size, mean age, percentage male, duration of illness). The tau squared (τ^2^) test and the *P* value are used to qualitatively analyses statistical heterogeneity between included studies. The larger the tau squared and the smaller the *P* value, the greater the likelihood of heterogeneity; conversely, the less heterogeneity exists. In addition, *I*^2^ is a parameter that quantifies the heterogeneity between the results of the studies. Its value is distributed from 0% to 100%. When *I*^2^ is less than 25%, it indicates low heterogeneity; 25% to 50% indicates moderate heterogeneity; and *I*^2^ > 75% indicates high heterogeneity. When *I*^2^ > 50%, this means that there is significant heterogeneity. We used both global and local methods to test for inconsistency in the study results. For global inconsistency, we assessed the inconsistency statistically using a test designed according to the treatment method.^[29]^ The node-splitting analysis was used to divide the mixed evidence into direct and indirect evidence to assess the consistency of the model.^[30]^ Summary SMDs, 95% confidence intervals (CI) or ORs, 95% CI for all two-way comparisons are presented using the league table. We ranked different interventions using surfaces under the cumulative ranking curve (SUCRA). In addition, we used a comparative adjusted funnel plot to assess potential publication bias. If the *P* value of the Egger test was less than .05, we considered the funnel plot to be asymmetric and subject to publication bias.

## 3. Results

### 3.1. Study selection

**Figure 1.**
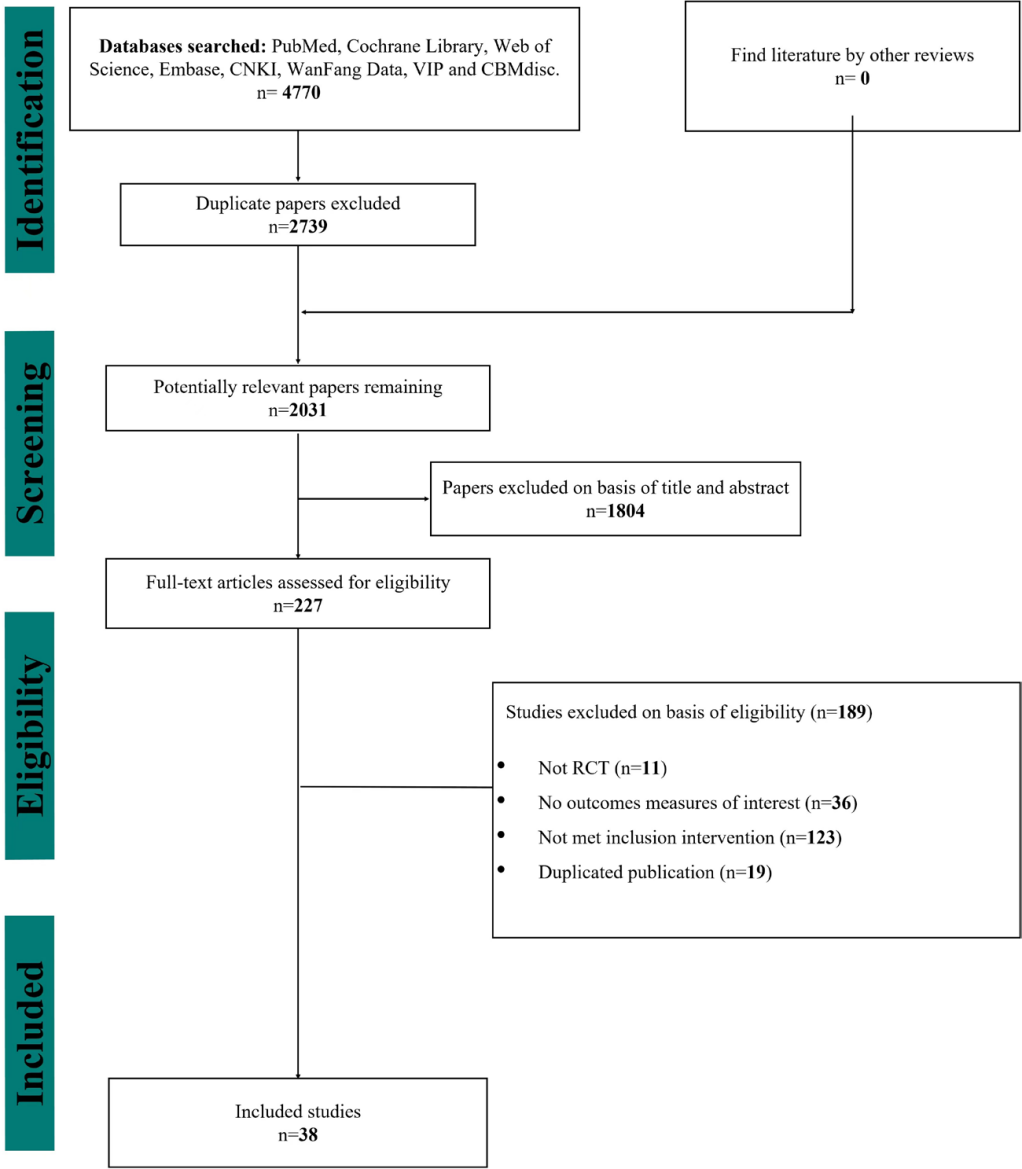
Flow diagram of study inclusion. RCT = randomized controlled trial.

An initial total of 4770 relevant studies were retrieved. After removing duplicates, 2031 remained. By reading the titles and abstracts, 1804 irrelevant studies including reviews, retrospective studies, and animal studies were removed. Ultimately, after reading the full text, the final 38 RCTs were included in this study. The literature screening process is shown in Figure 1.

### 3.2. Study characteristics

Thirty-eight RCTs published between 2007 and 2022, with sample sizes ranging from 60 to 316, corresponding to 4257 participants, were selected for pooled analysis. A total of 11 interventions including governor meridian moxibustion (GM), indirect moxibustion (IM), heat-sensitive moxibustion (HSM), dog day moxibustion (DDM), vesiculating moxibustion (VM), moxibustion alone (MOX), warming needle moxibustion (WNM), thunder fire moxibustion (TFM), combination therapy, conventional treatment (CVT), and placebo therapy (PT). The 38 RCTs included 35 two-armed studies and 3 three-armed studies. The characteristics of the included studies are shown in Supplementary File Table S3, Supplemental Digital Content, http://links.lww.com/MD/I503.

### 3.3. Quality assessments of studies

**Figure 2.**
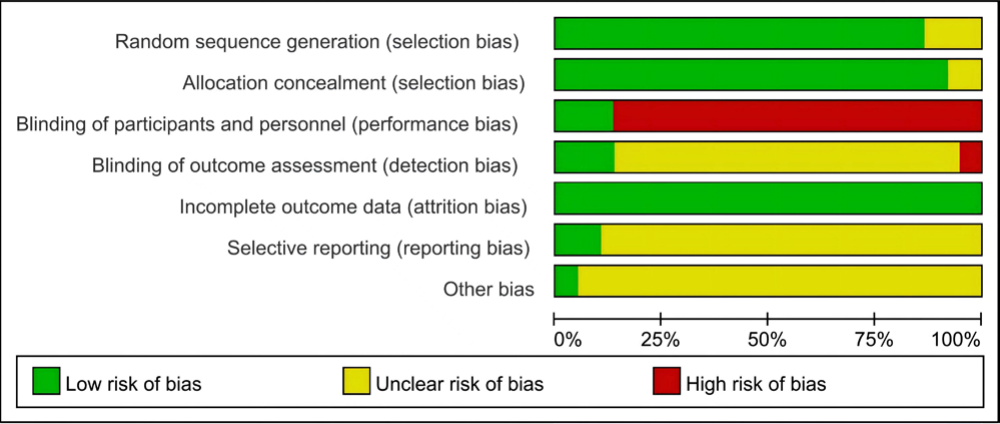
Network meta-analysis of eligible comparisons.

Most RCTs use a table of random numbers in the random sequence generation process and are therefore evaluated as low risk. 5 RCTs^[31–35]^ did not describe the particular randomization method evaluated as uncertain risk of bias. 3 RCTs^[36–38]^ were evaluated as low risk due to the use of the central allocation method. In terms of blinding, the 5 RCTs^[36–40]^ were blinded to investigator, patient, and outcome assessor so were evaluated as low risk. The presence or absence of other biases in the vast majority of included studies could not be clearly determined and they were therefore exposed to an uncertain risk of bias. (Fig. 2).

### 3.4. Results of the network meta-analysis

#### 3.4.1. Clinical effective rate.

**Figure 3.**
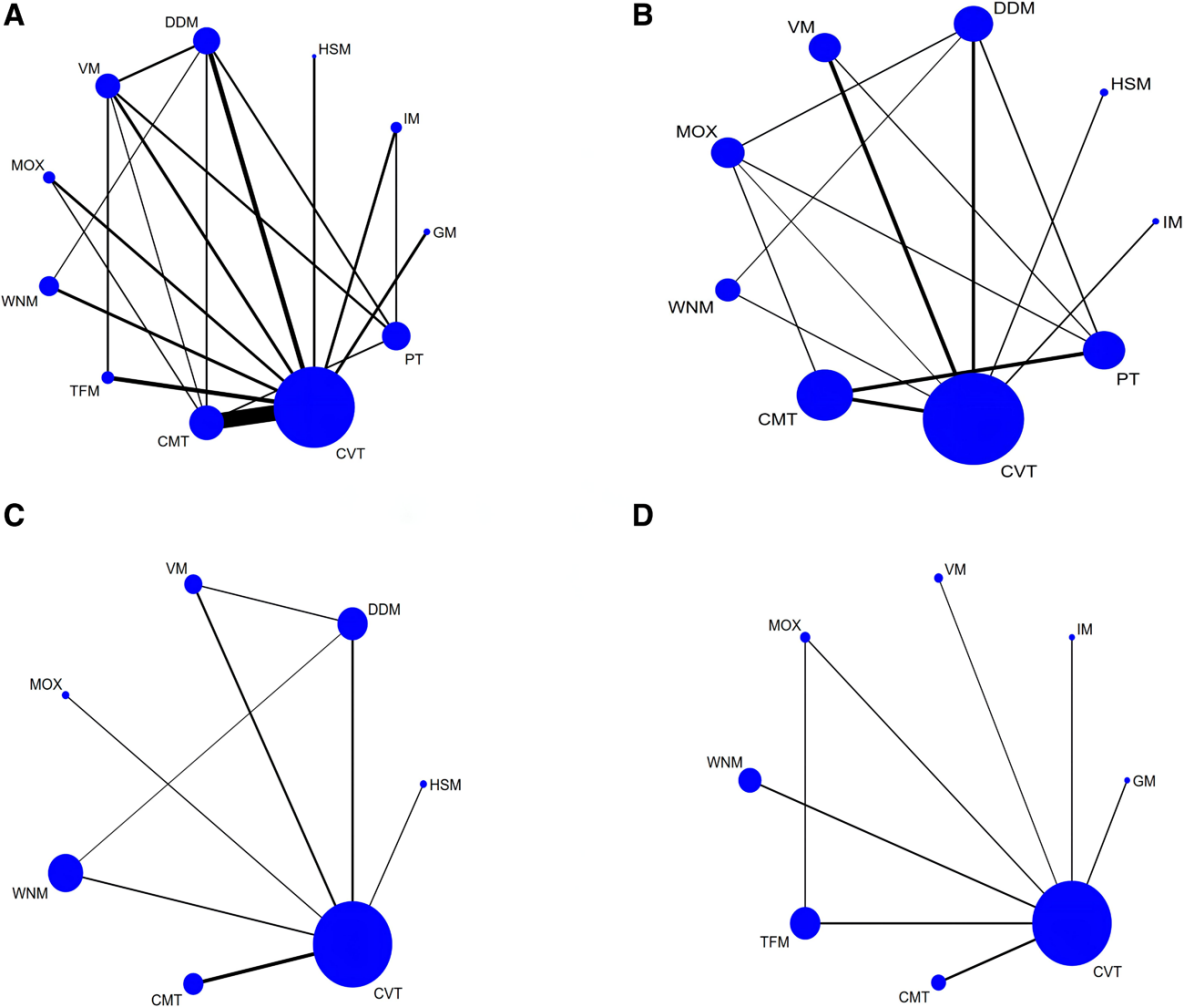
Network plots for different outcomes. (A) Clinical effective rate, (B) RQLQ scores, (C) IgE, (D) VAS scores. CVT = conventional treatment, DDM = dog day moxibustion, GM = governor meridian moxibustion, HSM = heat-sensitive moxibustion, IgE = immunoglobulin E, IM = indirect moxibustion, MOX = moxibustion alone, RQLQ = rhino-conjunctivitis quality of questionnaire, TFM = thunder fire moxibustion, VAS = visual analog scale, VM = vesiculating moxibustion, WNM = warming needle moxibustion.

**Figure 4.**
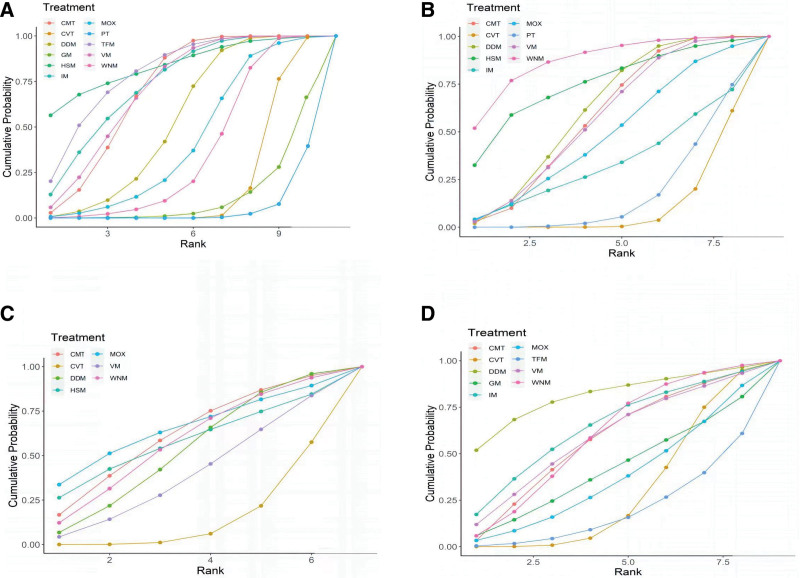
Surface under the cumulative ranking curve (SUCRA) plots for different outcomes. The vertical axis represents cumulative probabilities and the horizontal axis represents rank. (A) Clinical effective rate, (B) RQLQ scores, (C) IgE, (D) VAS scores. CVT = conventional treatment, DDM = dog day moxibustion, GM = governor meridian moxibustion, HSM = heat-sensitive moxibustion, IgE = immunoglobulin E, IM = indirect moxibustion, MOX = moxibustion alone, RQLQ = rhino-conjunctivitis quality of questionnaire, TFM = thunder fire moxibustion, VAS = visual analog scale, VM = vesiculating moxibustion, WNM = warming needle moxibustion.

A total of 35 studies^[31,34,38,39,41–53]^ (92%) with 4001 participants reported usable results for clinical effective rate (Fig. 3). There was a total of 11 interventions, including 9 moxibustion-related therapies, conventional treatment and placebo. IM, HSM, DDM, VM, MOX, TFM, and CMT significantly improved clinical effectiveness compared to placebo, with OR ranged between 32.77 (1.86 95% credible interval [Crl] to 1360.2) for HSM to 5.32 (1.05–27.04) for MOX. (Table 1). IM, HSM, DDM, VM, TFM, and CMT significantly improved clinical effectiveness compared to conventional treatment with the OR ranged between 14.34 (1.02–506.1) for HSM to 3.45 (95% CI 1.34–9.07) for DDM (Table 2). In addition, IM, VM, HSM, TFM, CVT significantly improved clinical effectiveness compared to GM. (Table 1). See Figure 4 for ranking probabilities of effectiveness rates. HSM (84.2%) had the best efficacy.

**Table 1:** Network meta-analysis result for clinical effective rate and RQLQ scores.

PT	0.07 (−0.31, 0.41)	NA	−0.09 (−0.9,0.71)	0.07(0.31, 0.41)	−0.34 (−0.73, 0.02)	0.31 (−0.09,0.76)	−0.24 (−0.83, 0.29)	0.67 (0.05, 1.3)	NA	−0.32 (−0.63,−0.06)
2.28 (0.76, 7.09)	CVT	NA	0.15 (−0.55, 0.85)	**0.6 (0.07, 1.29**)	**0.41 (0.08, 0.72**)	**0.38 (0.04, 0.72**)	0.32 (−0.16, 0.78)	**0.74 (0.18, 1.29**)	NA	**−0.39 (−0.72, −0.06**)
1.41 (0.21, 8.95)	0.61 (0.13, 2.76)	GM	NA	NA	NA	NA	NA	NA	NA	NA
**14.23 (3.37, 64.33**)	**6.24 (1.71, 24.29**)	**0.1 (0.01, 0.7**)	IM	−0.44 (−1.45, 0.51)	−0.26 (−1.01, 0.54)	0.22 (−0.56, 1.02)	0.15 (−0.7, 1.01)	0.58 (−0.32, 1.48)	NA	−0.23 (−1.03, 0.55)
**32.77 (1.86, 1360.2**)	**14.34 (1.02, 506.1**)	**0.04 (0, 0.91**)	0.4 (0.01, 8.4)	HSM	0.19 (−0.53, 0.93)	−0.22 (−0.98, 0.53)	−0.29 (−1.12, 0.52)	0.13 (−0.77, 0.99)	NA	0.21 (−0.55, 0.97)
**7.82 (2.8, 23.1**)	**3.45 (1.34, 9.01**)	0.18 (0.03, 1.05)	1.83 (0.41, 8.34)	4.2 (0.24, 172.7)	DDM	−0.03 (−0.46, 0.42)	−0.1 (−0.59, 0.4)	0.32 (−0.22, 0.9)	NA	0.03 (−0.37, 0.41)
**12.69 (4.16, 37.54**)	**5.53 (2, 15.37**)	**0.11 (0.02, 0.7**)	1.12 (0.25, 5.61)	2.58 (0.16, 108.2)	0.62 (0.2, 2.03)	VM	0.07 (−0.49, 0.66)	0.35 (−0.28, 1)	NA	−0.01 (−0.44, 0.41)
**5.32 (1.05, 27.14**)	2.32 (0.68, 8.17)	0.26 (0.04, 1.8)	2.68 (0.45, 16.73)	6.17 (0.31, 262.2)	1.48 (0.33, 6.71)	2.38 (0.5, 11.15)	MOX	0.42 (−0.29, 1.12)	NA	−0.07 (−0.64, 0.47)
4.17 (0.93, 19.7)	1.83 (0.61, 5.68)	0.33 (0.05, 2.17)	3.39 (0.62, 19.23)	7.84 (0.42, 327.7)	1.88 (0.5, 7.27)	3.03 (0.7, 12.56)	1.27 (0.23, 6.65)	WNM	NA	0.35 (−0.28, 0.95)
**17.9 (3.54, 89.38**)	**7.77 (2.29, 27.61**)	**0.08 (0.01, 0.55**)	0.81 (0.14, 5)	1.85 (0.1, 77.87)	0.44 (0.09, 2.04)	0.71 (0.15, 3.22)	0.3 (0.05, 1.7)	0.23 (0.04, 1.27)	TFM	NA
**12.21 (3.97, 39.83**)	**5.37 (2.75, 10.67**)	**0.11 (0.02, 0.59**)	1.17 (0.28, 4.93)	2.67 (0.17, 96.23)	0.64 (0.23, 1.82)	1.03 (0.34, 2.96)	0.43 (0.12, 1.56)	0.34 (0.09, 123)	1.45 (0.36, 59)	CMT

Significant result is in bold.

CVT = conventional treatment, DDM = dog day moxibustion, GM = governor meridian moxibustion, HSM = heat-sensitive moxibustion, IM = indirect moxibustion, MOX = moxibustion alone, PT = placebo therapy, RQLQ = rhino-conjunctivitis quality of questionnaire, TFM = thunder fire moxibustion, VM = vesiculating moxibustion, WNM = warming needle moxibustion.

**Table 2:** Network meta-analysis result for IgE and VAS scores.

CVT	NA	NA	0.27 (−0.43, 1)	0.22 (−0.18, 0.69)	0.13 (−0.31, 0.6)	0.34(−0.42, 1.06)	0.27 (−0.21, 0.74)	NA	−0.3(−0.67, 0.18)
0.08 (−0.82, 1.01)	GM	NA	NA	NA	NA	NA	NA	NA	NA
0.4 (−0.5, 1.28)	0.31 (−1.04, 1.58)	IM	NA	NA	NA	NA	NA	NA	NA
NA	NA	NA	HSM	0.04 (−0.8, 0.87)	−0.13 (−0.99, 0.74)	0.07 (−0.98, 1.1)	0 (−0.86, 0.85)	NA	−0.03 (−0.81, 0.87)
0.73 (−0.45, 1.95)	−0.66 (−2.16, 0.86)	−0.34 (−1.93, 1.11)	NA	DDM	−0.09 (−0.62, 0.41)	0.12 (−0.78, 0.91)	0.04 (−0.52, 0.57)	NA	−0.08 (−0.61, 0.59)
0.29 (−0.57, 1.16)	0.2 (−1.05, 1.44)	−0.11 (−1.3, 1.2)	NA	−0.45 (−1.94, 1.06)	VM	−0.19 (−1.03, 0.69)	0.13 (−0.52, 0.77)	NA	−0.15 (−0.74, 0.52)
0.05 (−0.65, 0.83)	−0.03 (−1.18, 1.12)	−0.34 (−1.45, 0.85)	NA	−0.69 (−2.06, 0.81)	0.24 (−0.96, 1.39)	MOX	−0.07 (−0.94, 0.8)	NA	0.04 (−0.76, 0.91)
0.3 (−0.27, 0.94)	0.21 (−0.84, 1.29)	−0.1 (−1.14, 1.01)	NA	−0.44 (−1.75, 0.9)	0.01 (−1.02, 1.05)	0.25 (−0.72, 1.17)	WNM	NA	−0.03 (−0.64, 0.64)
−0.15 (−0.8, 0.47)	−0.25 (−1.37, 0.89)	−0.56 (−1.65, 0.58)	NA	−0.89 (−2.3, 0.52)	0.45 (−0.62, 1.52)	−0.2 (−1.06, 0.56)	0.47 (−0.4, 1.35)	TFM	NA
−0.31 (−1.14, 0.52)	0.08 (−1.17, 1.27)	−0.22 (−1.47, 0.98)	NA	0.43 (−0.45, 1.3)	−0.02 (−1.22, 1.2)	−0.27 (−1.32, 0.9)	−0.01 (−1, 1.04)	−0.47 (−1.51, 0.63)	CMT

CVT = conventional treatment, DDM = dog day moxibustion, GM = governor meridian moxibustion, HSM = heat-sensitive moxibustion, IgE = immunoglobulin E, IM = indirect moxibustion, MOX = moxibustion alone, TFM = thunder fire moxibustion, VAS = visual analog scale, VM = vesiculating moxibustion, WNM = warming needle moxibustion.

#### 3.4.2. RQLQ scores.

A total of 18 studies^[19,31,32,36–40,44,46–48,52,54–58]^ (47%) with 1823 participants reported usable results for RQLQ scores (Fig. 3). There was a total of 9 interventions, including 7 moxibustion-related therapies, conventional treatment and placebo. DDM, VM, HSM, WNM, and CMT significantly reduced RQLQ scores compared to conventional treatment with SMDs ranged between −0.39 (95% CI −0.72 to −0.06) for CMT to 0.74 (95% CI 0.18–1.29) for WNM (Table 1). WNM, HSM, and CMT significantly reduced RQLQ scores compared to placebo with SMDs ranged between −0.32 (95% CI −0.63 to −0.06) for CMT to 0.67 (95% CI 0.05–1.3) for WNM (Table 1). See Figure 4 for ranking probabilities of RQLQ scores. WNM (87.5%) had the best effect in reducing RQLQ scores.

#### 3.4.3. IgE.

Only 10 studies^[17,32,33,38,43,44,47,52,56,59]^ (26%) with 1047 participants presented usable results for serum IgE levels (Fig. 3). There was a total of 7 interventions, including 6 moxibustion-related therapies and conventional treatment. In the network meta-analysis, there were no significant differences in the six moxibustion-related therapies compared to the control group for each comparison (Table 2). See Figure 4 for ranking probabilities of IgE. Based on ranking analysis, MOX (65%) takes first rank.

#### 3.4.4. VAS scores.

Only 8 studies^[43,45,46,53,58,60–62]^ (21%) with 905 participants presented usable results for VAS scores (Fig. 3). There was a total of 9 interventions, including 8 moxibustion-related therapies and conventional treatment. In the network meta-analysis, there were no significant differences in the eight moxibustion-related therapies compared to the conventional treatment for each comparison (Table 2). See Figure 4 for ranking probabilities of VAS scores. DDM (81.9%) had the best effect in reducing VAS scores.

#### 3.4.5. ADR events.

Among 38 RCTs, a total of 7 RCTs (18.4%) reported adverse effects. Adverse events mainly include rash, pruritus, blisters and dry mouth. The ADR criteria were not entirely consistent in each RCT, so only descriptive analyses were performed. Given the small sample size, it is not yet possible to prove that the differences between the two groups were statistically significant. Please see Table 3 for detailed information.

**Table 3:** Summary of adverse events.

Type of interventions	Number of RCTs	Groups	Total sample size	Incidence (%)	Detailed ADR events (number of cases)
VM vs PT	1	VM	46	42.9	Rash (17 occurrences in 4 wk), pruritus (23 occurrences in 4 wk), blisters (3 occurrences in 4 wk), pigmentation (36 occurrences in 4 wk)
		PT	44	0.08	Rash (3 occurrences in 4 wk) 3, pruritus (7 occurrences in 4 wk) 7, blisters (1occurrences in 4 wk), pigmentation (4 occurrences in 4 wk)
VM vs CVT	1	VM	41	0.09	Flushed skin 2 pruritus 2
		CVT	38	10.5	Sleepiness and dry mouth 4
WNM vs CVT	1	WNM	105	0.02	Halo needle 1 fear of needle 1 allergy to moxa smoke 1
		CVT	105	0.02	Headache 1 dry mouth 1 stomachache 1
CMT vs PT	1	CMT	32	0.01	Allergy to moxa smoke 1
		PT	32	none	–
CMT vs CVT	1	CMT	41	0.02	Blisters 1
		CVT	38	none	–
GM vs CVT	1	GM	28	none	–
		CVT	29	10.3	Dryness of the nasal passages 1 bitterness in mouth 1 dizziness 1
MOX vs CVT	1	MOX	90	11.1	Skin burns 6 skin allergy 4
		CVT	90	33.3	Dry nose 25 nasal congestion 5

CVT = conventional treatment, GM = governor meridian moxibustion, MOX = moxibustion alone, PT = placebo therapy, RCT = randomized controlled trial, VM = vesiculating moxibustion, WNM = warming needle moxibustion.

### 3.5. Heterogeneity and inconsistency assessment

Heterogeneity was assessed for clinical effective rate, RQLQ scores, IgE, and VAS scores. Heterogeneity in clinical effective rate and IgE was assessed as moderate to high. Heterogeneity in RQLQ scores and VAS scores was assessed as moderate. We tested the inconsistency of all comparisons of clinical effective rate, RQLQ, IgE, and VAS. An assessment of local inconsistencies across all outcomes showed that most loops were consistent. Only three comparisons (2.1%) had a significant difference in clinical effective rate. All the results are presented in the Supplementary File Table S4 and Table S5, Supplemental Digital Content, http://links.lww.com/MD/I504.

### 3.6. Small-study effect analysis

**Figure 5.**
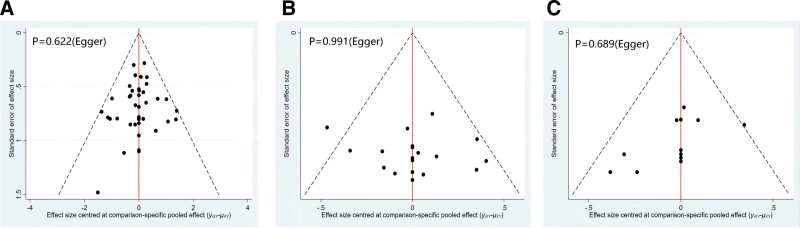
Comparison-adjusted funnel plots (A) clinical effective rate, (B) RQLQ scores, (C) IgE. IgE = immunoglobulin E, RQLQ = rhino-conjunctivitis quality of questionnaire.

Comparison-adjusted funnel plots suggested that there may be no small study effect. (Egger test; *P* > .05) (Fig. 5). Because there are fewer than ten studies using VAS scores as the outcome indicator, no funnel plot was performed.

## 4. Discussion

In this study, 38 RCT of moxibustion treatment for AR were included, and including 4257 patients and 9 moxibustion-related treatment modalities. The main results were as follows: HSM had the best efficacy in terms of effective rate, In addition, IM, VM, TFM, and CMT were also significantly better than CVT, PT, and GM. WNM had the best effect in reducing RQLQ scores. In addition, HSM and CMT were also better than CVT and PT. In terms of IgE and VAS scores, there was no significant difference between moxibustion and conventional treatment. 7 RCTs have reported minor adverse reactions. All the adverse reactions could be alleviated spontaneously without causing serious adverse reactions.

In terms of clinical efficacy, HSM has the best effect in the treatment of AR. HSM is a new moxibustion method; different from the traditional moxibustion, HSM has selected heat-sensitive points to treat diseases. Researchers have found that some acupoints in the human body are extremely sensitive to moxibustion-heat under pathological conditions, these acupoints are called heat-sensitive points, and the location of heat-sensitive points will change with the development of the disease. Stimulation of these acupoints will produce heat-sensitive reactions (i.e., comfortable thermal inductance such as thermal transmission, thermal diffusion, thermal penetration, feverish limbs, body heat, gastrointestinal peristalsis, and skin spreading flushing); heat-sensitive phenomena is considered to be the key to improve the efficacy of moxibustion.^[63,64]^ Basic research has confirmed the existence of a “heat-sensitive reaction phenomenon.”^[65]^ In addition, the effect of IM, VM, TFM, and CMT is not only better than PT, but also better than CVT and GM. At present, from the perspective of traditional Chinese medicine, AR is formed in the deficiency of vital energy of the lung, spleen and kidney, which makes the Yang Qi of the nasal cavity insufficient. Moxibustion in the treatment of AR will produce warm and thermal stimulation to act on meridians and acupoints, which can achieve the effect of “supplementing with warmth” through its warming and tonifying effect, that is, strengthening Yang Qi and tonifying Qi.^[66]^ Both IM and VM use drugs with warm flavor during moxibustion.^[33,67]^ The warm and heat stimulation of moxibustion and the effect of medicine warming yang and benefiting Qi can cooperate with each other to better exert its warming and tonifying effect. Compared with other moxibustion, TFM is stronger, with large fire power and sufficient moxibustion volume, the fire head temperature can reach 240°C, more than twice that of ordinary moxibustion. Therefore, the warm force generated by TFM can penetrate through the skin and reach the viscera, playing the role of warming Yang, helping fire and diffusing the nasal cavity.^[68]^ A previous study also confirmed that TFM is safe and effective in the treatment of AR.^[17]^ CMT is a comprehensive treatment method, which combines moxibustion with other traditional Chinese medicine treatments to treat AR, so as to exert the maximum complementary effect of 1 + 1 > 2 and make the final treatment effect significantly better than that of monotherapy. In recent years, with the continuous progress of moxibustion research, our understanding of the mechanism of moxibustion therapies for AR has been further deepened. There are mainly three mechanisms: 1. Regulate the immune system. Studies have found that HSM can reduce the content of pro-inflammatory cytokines (IL-4, IL-5, IL-13) related to helper T cell 2 (Th2) and increase the content of anti-inflammatory cytokines (IL-12, IFN-γ) related to helper T cell 1(Th1) and reduce the secretion of related inflammatory cytokines in the nasal mucosa of rats. Thus, improving the AR symptoms.^[69]^ IM can improve the serum hemolysase level of mice and increase the secretion of anti-inflammatory cytokine 1L-2, thereby improving AR symptoms. 2. Reduce the infiltration of inflammatory cells. Jin’s study confirmed that VM can down-regulate the expression of NF-KB in AR rats, and reduce the release of inflammatory cytokines, thus exerting anti-inflammatory effects.^[70]^ 3. Regulate water metabolism. It has been proven that CMT can reduce the high expression of aquaporin AQP1, AQP2, and AQP5 mRNA in nasal mucosa of AR rat model to regulate water metabolism and thus alleviate the symptoms of AR rats.^[71]^

Our results show that WNM performs best in reducing RQLQ scores. Acupuncture at specific acupoints can effectively relieve nasal symptoms such as nasal congestion.^[72]^ By combining acupuncture and moxibustion, WNM has the dual effect of acupuncture and moxibustion at the same time, and when acupuncture and moxibustion are used together, it is both heat transfer and sensing, and the effect of improving symptoms is more lasting than that of acupuncture. Therefore, compared with other moxibustion types, WNM is more likely to maximize the efficacy in improving the quality of life of patients. Studies have found that WNM can reduce inflammation reaction by regulating the nervous system and immune system, improve the cellular immune function of the body, inhibit allergy reaction, enhance the immunity of patients, and thus improving the quality of life of patients.^[73]^ In addition, WNM will generally increase the acupoints for regulating emotions when selecting points, and alleviating emotions is more conducive to improving the quality of life of patients. HSM can stimulate the special regulation system of human body through diathermy, heat expansion and heat transfer, to improve the overall physique of patients and better relieve the nose and eye symptoms and sleep quality.^[74]^ CMT can also significantly reduce RQLQ scores. In clinical practice, moxibustion is often combined with acupuncture, traditional Chinese medicine, acupuncture and cupping, acupoint catgut embedding and other traditional Chinese medicine treatments to achieve better therapeutic effects. Although the clinical manifestations of AR are mainly nasal symptoms, some patients have individual differences such as insomnia, headache and fatigue.^[75]^ Acupuncture, traditional Chinese medicine and other treatments can give personalized treatment to patients according to symptoms on the basis of syndrome differentiation and treatment and improve the quality of life of patients. Combination therapies can make up for the deficiency of monotherapy, resulting in better effects and shorter treatment time.^[76]^

There was no significant difference between moxibustion and conventional therapy in reducing IgE level and VAS score. Sun found that moxibustion and Western medicine can improve VAS score and regulate serum IgE. Although this study did not reflect the significant advantages of moxibustion in improving VAS score and regulating serum IgE in AR patients, some studies have found that moxibustion combined with western medicine has more advantages in improving VAS score and regulating serum IgE in AR patients than western medicine alone.^[77]^

The adverse reactions of moxibustion therapy, such as skin damage of patients, are mainly concentrated in VM, which is related to the characteristics of VM, apply the medicine with irritation to the skin to the acupoint or the affected area, and the disease is treated by natural congestion, flushing or blistering of the local skin. A small number of patients are allergic to moxa smoke, which may be related to the patient’s personal constitution. There are also a few patients with slight skin burns, which suggests that clinicians should pay attention to the operation specifications during moxibustion.

Although this study compared the efficacy of different moxibustion related therapies in the treatment of AR through network meta-analysis, there are still some limitations in this study. First, most RCTs did not report allocation concealment, blinding and registration, which may weaken the reliability and robustness of our findings. Secondly, each moxibustion method has different acupoint selection, operation technique and moxibustion amount, and these factors are potentially inconsistent. Then, all the studies are conducted in China, and all the study participants are Chinese, meaning the results of this study may not be applicable to patients in other countries. Finally, of the 38 included studies, only 10 and 8 reported IgE and VAS, respectively. It is hoped that more reported VAS and IgE results will be added to future trials to comprehensively evaluate the treatment effect.

## 5. Conclusion

In conclusion, HSM may be the most effective treatment for AR. Compared with other types of moxibustion, HSM has a higher clinical efficiency and can also improve the quality of life of AR patients, which may be the best choice for AR patients in moxibustion treatment. Therefore, HSM can be used for AR patients who are not ideal for traditional drug treatment or cannot tolerate adverse drug reactions.

## Author contributions

**Data curation:** Ke Chen, Chaofeng Hou.

Writing – original draft: Ke Chen.

Writing – review & editing: Liu Chengjiang, Yong Meng.

## Supplementary Material










