# Supplementary material for: Efficacy comparison of different moxibustion treatments for allergic rhinitis: A systematic review and Bayesian network meta-analysis
Source: Medicine (Baltimore). 2023 Mar 3;102(9):e32997. doi: 10.1097/MD.0000000000032997 (PMC9981372; doi:10.1097/MD.0000000000032997)
Supplement: Supplementary file 2 [file medi-102-e32997-s002.pdf]

Table S1:

Search Strategy in PubMed

Search	Query
#1	Moxibustion [Mesh]
#2	Moxabustion [Title/Abstract]
#3	Moxa [Title/Abstract]
#4	Mugwort [Title/Abstract]
#5	#1 or #2 or #3 or #4
#6	Rhinitis, Allergic [Mesh]
#7	Allergic Rhinitides[Title/Abstract]
#8	Rhinitides, Allergic[Title/Abstract]
#9	Allergic Rhinitis[Title/Abstract]
#10	#10 #6 OR #7 OR #8 OR #9
#11	#11 #5 and #10
