# Supplementary material for: Efficacy comparison of different moxibustion treatments for allergic rhinitis: A systematic review and Bayesian network meta-analysis
Source: Medicine (Baltimore). 2023 Mar 3;102(9):e32997. doi: 10.1097/MD.0000000000032997 (PMC9981372; doi:10.1097/MD.0000000000032997)
Supplement: Supplementary file 3 [file medi-102-e32997-s003.pdf]

Table S2 : Definition of different moxibustion therapies

Type of moxibustion	Defifinitions
GM （governor meridian moxibustion）	A special method of moxibustion applied to the spinal column of the Governor's Vessel for the treatment of disease and health.
IM (indirct moxibustion)	Indirect moxibustion, also known as spacer moxibustion or interval moxibustion, is a method of using other medicines to separate the moxa cones from the acupuncture points
HSM (heat-sensitive moxibustion)	Heat-sensitive moxibustion is a form of moxibustion suspension therapy that uses moxa sticks to apply moxibustion to heat-sensitive points on the body.
DDM (dog day moxibustion)	Dog day moxibustion refers to moxibustion therapy on the day of three volts, which is generally chosen to be performed on the day of three volts
VM (vesiculating moxibustion)	It is a treatment method that uses skin-irritating drugs applied to acupuncture points or affected areas to make the local skin naturally congested, flushed or blistered.
MOX (moxibustion alone)	Moxibustion is a type of external treatment in Chinese medicine, which relies on the burning of moxa or moxa sticks to produce a warm sensation, acting on the meridians and acupuncture points on the surface of the skin, thus exerting a therapeutic effect.
WNM (Warming needle moxibustion)	Warming needle moxibustion is a type of moxibustion, which refers to making small sections of moxa cones after needling, inserting the cones into the needle handles, and then lighting the cones to allow the heat to enter the body through the body of the needles.
TFM (thunder fire moxibustion)	Thunder fire moxibustionis a moxibustion method that uses herbal powder and moxa wool to make moxa strips, which are applied to acupuncture points.
CMT （Combination therapy）	Combination therapy refers to the combination of two groups of moxibustion methods, or one of the above moxibustion therapies combined with other traditional Chinese medicine therapies (e.g. herbs, herbal injections, acupuncture, etc.)
