# Supplementary material for: Efficacy comparison of different moxibustion treatments for allergic rhinitis: A systematic review and Bayesian network meta-analysis
Source: Medicine (Baltimore). 2023 Mar 3;102(9):e32997. doi: 10.1097/MD.0000000000032997 (PMC9981372; doi:10.1097/MD.0000000000032997)
Supplement: Supplementary file 4 [file medi-102-e32997-s004.pdf]

Table S3 : Characteristics of the studies included in this meta-analysis

Study ID	N(E/C)	Sex(M/F)	Age (years)	Treatment group	Control group	Course (days)	Outcomes
Liang 2019	46/46	48/44	T:45.6±15.1<br>C:40.4±15.6	VM	PT	28	2
Lin 2014	72/78	72/78	T:21±4;<br>C:21±4	DDM	CVT	43	1
Ding 2016	53/48	49/54	T:41±12;<br>C:43±11	GM	CVT	90	1
Qin 2020	105/105	97/98	T:40±20;<br>C:40±19	IM	CVT	90	1
Ke 2014	42/40	39/43	T:36±1<br>C:38±19	CMT	CVT	30	1,2
Xie 2015	78,77/76	113/118	T1:39±11;<br>T2:36±12<br>C:39±12	DDM<br>MOX	CVT	2 years	1,2,3
Sun 2020	105/105	116/94	T:38.25±8.839<br>C:36.52±9.86	WNM	CVT	28	1,3,4
Ting 2019	32/32	32/32	T:43.59±13.67<br>C:41.81±14.80	CMT	PT	28	2
Fang 2014	34/38	35/37	T:32±8<br>C:35±8	DDM	MOX	60	1.2,3
Xu 2021	28/29	18/39	T:36.25±10. 55<br>C:34. 0±11. 21	GM	CVT	28	1,4
Luo2021	65/65	70/60	T:36.83±9.01<br>C:37.55±9.47	CMT	CVT	28	1,2,4
Huang2018	45;45/45	65/59	T1:30.15±12.18;<br>T2:29.70±12.36<br>C:30.50±13.87	CMT<br>MOX	PT	30	1,2
Wang2020	40/40	27/53	T:29±12<br>C:28±12	HSM	CVT	180	1,2,3
Zhu 2017	32/30	32/30	T:42.75±13.04<br>C:40.67± 12.70	DDM	CVT	28	1,2,3
Deng 2012	28/26	27/27	T:28.36±13.35<br>C:33.1±916.71	DDM	PT	50	1,2
Zhang2021	90/90	93/87	T:46±15<br>C:48±15	Mox	CVT	28	1,2
Li 2020	30/30	31/29	T35.90±8.94<br>C;36. 60±9. 07	DDM	CVT	30	1
Zhao 2019 [	39/39	38/40	T:32.27± 2.36<br>C:32.31 ±2.18	CMT	CVT	21	1
Wu 2019	36/36	31/41	T:47.22±4.64<br>C:45.45±4.92	CMT	CVT	21	1,

Study ID	N(E/C)	Sex(M/F)	Age (years)	Treatment group	Control group	Course (days)	Outcomes
Wang 2016	30/30	29/31	T:18-52<br>C:20-55	CMT	CVT	30	1,2,3
Cheng2018	44/42	44/42	T:33.43±9.13<br>C:33.43±9.13	MOX	CVT	23	1,2,3
Ma 2018	30/30	21/39	T:36.9±10.66;<br>C:40.17±12.32	IM	PT	60	1
Wang 2019	37/37	36/38	T: 15 -53<br>C:16-52	CMT	MOX	30	1
Sun 2019	52/52	57/43	T:38.70± 9.15<br>C:36.5± 9.86)	WNM	CVT	28	1,4
Shou 2022	79/81	81/79	T:40±11<br>C:37±11	CMT	PT	28	1,2
Pan 2021	73/73	79/67	T:31.36±3.42<br>C:32.13±2.64	CMT	CVT	28	1,2,3
Tang 2021	50/50	30/70	T:28.76±1.53<br>C:27.16±0.94	VM	CVT	28	2,3,4
Ma 2020	31/31	30/32	T:41.87±4.90<br>C:42.23±4.28	CMT	DDM	50	1,4
Li 2018	61/60	51/70	T:48±12<br>C:40±11	CMT	CVT	2 years	1,2
Zhang2017	161/81	120/122	T:40±11<br>C:41±12	DDM	PT	30	1
Liu 2020	32/48	52/28	T:36.97±5.16<br>C:37.07±5.12	VM	CVT	42	1,3
Yu 2017	35/35	28/35	T:37±4<br>C:37±4	CMT	CVT	28	1
Jiang 2022	42/42	39/45	T:35.1± 4.9<br>C:35.3± 4.7	CMT	VM	28	1
Wang 2019	30,30/30	29/61	T1:32.87±11.10<br>T2:32.77±11.65<br>C:32.80±10.61	CMT	CVT	21	1,4
Mi 2011	158/158	156/160	T:36±12<br>C:36±13	VM	PT	90	1
Zhao 2007	60/60	59/61	-	TFM	CVT	21	1
Zhao 2017	51/51	67/35	T:45.33±1.39<br>C:45.29±1.35	TFM	CVT	21	1
Zhuang2021	30/30	25/34	T:35.43±11.45<br>C:37.24±12.29	IM	CVT	20	1,2,4
