# Supplementary material for: Efficacy comparison of different moxibustion treatments for allergic rhinitis: A systematic review and Bayesian network meta-analysis
Source: Medicine (Baltimore). 2023 Mar 3;102(9):e32997. doi: 10.1097/MD.0000000000032997 (PMC9981372; doi:10.1097/MD.0000000000032997)
Supplement: Supplementary file 5 [file medi-102-e32997-s005.pdf]

Table S4:**assessment of heterogeneity.**

Outcomes	Number of studies	Heterogeneity						SIDE	Splitting	the Design-by-treament-test			
		t^2	Q	df	P	I^2	Heterogeneity assessment	Number of insonsistent comparisons out of total	Percentage of insonsistent comparisons out of total	Q	df	t^2	P-value
Clinical effective rate	35	0.4746	58.97	27	0.0004	54.2%	moderate to high.	3	8%	22.12	11	0.0237	0.2177
RQLQ	17	0.0273	11.44	7	0.1205	38.8%	moderate	2	11%	4.86	4	0.0186	0.2988
IgE	10	0.0425	12.46	6	0.0525	51.8%	moderate to high.	1	10%	1.87	3	0.0770	0.5988
VAS	9	0.0273	3.31	2	0.1989	39.6%	moderate	1	11%	0	0	0.0273	-

Table S5:

assessment of Inconsistency.

comparison	k	prop	NMA		Direct		Indir		Diff		z	p
			TE	seTE	seTE	lower	TE	seTE	TE	seTE		
GM vs IM	0	0	2.136839729	0.880988053	NA	NA	2.136839729	0.880988053	NA	NA	NA	NA
GM vs HSM	0	0	2.715957076	1.453905086	NA	NA	2.715957076	1.453905086	NA	NA	NA	NA
GM vs DDM	0	0	1.532259117	0.795318095	NA	NA	1.532259117	0.795318095	NA	NA	NA	NA
GM vs VM	0	0	2.139702583	0.812581954	NA	NA	2.139702583	0.812581954	NA	NA	NA	NA
GM vs MOX	0	0	1.206939193	0.872132652	NA	NA	1.206939193	0.872132652	NA	NA	NA	NA
GM vs WNM	0	0	1.007089373	0.820031799	NA	NA	1.007089373	0.820031799	NA	NA	NA	NA
GM vs TFM	0	0	2.411331793	0.867360949	NA	NA	2.411331793	0.867360949	NA	NA	NA	NA
GM vs CMT	0	0	1.953904657	0.738182164	NA	NA	1.953904657	0.738182164	NA	NA	NA	NA
GM vs CVT	2	1	0.438689776	0.676255371	0.438689776	0.676255371	NA	NA	NA	NA	NA	NA
GM vs PT	0	0	-0.368749416	0.825397174	NA	NA	-0.368749416	0.825397174	NA	NA	NA	NA
IM vs HSM	0	0	0.579117347	1.405466931	NA	NA	0.579117347	1.405466931	NA	NA	NA	NA
IM vs DDM	0	0	-0.604580612	0.664253817	NA	NA	-0.604580612	0.664253817	NA	NA	NA	NA
IM vs VM	0	0	0.002862854	0.681049436	NA	NA	0.002862854	0.681049436	NA	NA	NA	NA
IM vs MOX	0	0	-0.929900536	0.785109254	NA	NA	-0.929900536	0.785109254	NA	NA	NA	NA
IM vs WNM	0	0	-1.129750356	0.724414441	NA	NA	-1.129750356	0.724414441	NA	NA	NA	NA
IM vs TFM	0	0	0.274492064	0.779666373	NA	NA	0.274492064	0.779666373	NA	NA	NA	NA
IM vs CMT	0	0	-0.182935072	0.622956492	NA	NA	-0.182935072	0.622956492	NA	NA	NA	NA
IM vs CVT	2	0.763520255	-1.698149953	0.564640259	-1.197428798	0.646191973	-3.314824331	1.161114013	2.117395533	1.328815193	1.59344621	0.111060126
IM vs PT	1	0.385531265	-2.505589145	0.646761682	-3.8066625	1.0416324	-1.689266967	0.825076821	-2.117395533	1.328815193	-1.59344621	0.111060126
HSM vs DDM	0	0	-1.183697959	1.353413543	NA	NA	-1.183697959	1.353413543	NA	NA	NA	NA
HSM vs VM	0	0	-0.576254493	1.36363	NA	NA	-0.576254493	1.36363	NA	NA	NA	NA
HSM vs MOX	0	0	-1.509017883	1.399933109	NA	NA	-1.509017883	1.399933109	NA	NA	NA	NA
HSMvs WNM	0	0	-1.708867703	1.368082416	NA	NA	-1.708867703	1.368082416	NA	NA	NA	NA
HSM vs TFM	0	0	-0.304625283	1.396965412	NA	NA	-0.304625283	1.396965412	NA	NA	NA	NA
HSM vs CMT	0	0	-0.762052419	1.320647664	NA	NA	-0.762052419	1.320647664	NA	NA	NA	NA
HSM vs CVT	1	1	-2.2772673	1.287058147	-2.2772673	1.287058147	NA	NA	NA	NA	NA	NA
HSM vs PT	0	0	-3.084706491	1.37130516	NA	NA	-3.084706491	1.37130516	NA	NA	NA	NA
DDM vs VM	1	0.155751866	0.607443466	0.511537179	-2.0084305	1.296165849	1.090035297	0.556726828	-3.098465797	1.41067029	-2.196449318	0.028059799
DDMvs MOX	0	0	-0.325319924	0.680564256	NA	NA	-0.325319924	0.680564256	NA	NA	NA	NA
DDMvs WM	1	0.440605958	-0.525169744	0.573981595	-0.89097292	0.864714585	-0.23704537	0.767430487	-0.65392755	1.156149154	-0.565608294	0.571660088
DDM vs TFM	0	0	0.879072676	0.676961939	NA	NA	0.879072676	0.676961939	NA	NA	NA	NA

DDM vs CMT	1	0.181395014	0.42164554	0.462178233	1.618096	1.085167594	0.156523593	0.510825178	1.461572407	1.199387789	1.218598705	0.222996541
DDM vs CVT	3	0.542817005	-1.093569341	0.418580392	-1.200888561	0.568135802	-0.966148379	0.619061573	-0.234740182	0.840247297	-0.279370351	0.779960622
DDM vs PT	2	0.584874508	-1.901008533	0.457374155	-1.57624085	0.598053827	-2.358577008	0.70987506	0.782336158	0.928219253	0.842835521	0.399320439
VM vs MOX	0	0	-0.93276339	0.697508946	NA	NA	-0.93276339	0.697508946	NA	NA	NA	NA
VM vs WNM	0	0	-1.13261321	0.630758048	NA	NA	-1.13261321	0.630758048	NA	NA	NA	NA
VM vs TFM	1	0.330561187	0.27162921	0.675581164	-0.44183275	1.175037163	0.623928521	0.825699626	-1.065761271	1.436137949	-0.742102297	0.458025358
VM vs CMT	1	0.280947134	-0.185797926	0.476685807	-1.3083328	0.899331733	0.252797024	0.562149476	-1.561129824	1.060570412	-1.47197188	0.141028471
VM vs CVT	2	0.425500237	-1.701012807	0.450519817	-1.606819409	0.690659509	-1.770776654	0.594386548	0.163957245	0.911211241	0.179933299	0.857204933
VM vs PT	2	0.582336651	-2.508451999	0.467300597	-2.639572851	0.612363468	-2.32563376	0.723074678	-0.313939091	0.947536811	-0.331321261	0.740401833
MOX vs WM	0	0	-0.19984982	0.718164497	NA	NA	-0.19984982	0.718164497	NA	NA	NA	NA
MOX vsTFM	0	0	1.2043926	0.772183124	NA	NA	1.2043926	0.772183124	NA	NA	NA	NA
MOX vs CMT	1	0.355632423	0.746965464	0.579889439	2.373681	0.972399048	-0.15083388	0.722401035	2.52451488	1.21137243	2.084012164	0.037159061
MOX vs CVT	2	0.708123085	-0.768249417	0.550721378	-1.505097032	0.654451638	1.019417848	1.019370501	-2.52451488	1.21137243	-2.084012164	0.037159061
MOX vs PT	0	0	-1.575688609	0.711817627	NA	NA	-1.575688609	0.711817627	NA	NA	NA	NA
WNM vs TFM	0	0	1.40424242	0.712777949	NA	NA	1.40424242	0.712777949	NA	NA	NA	NA
WNMvs CMT	0	0	0.946815284	0.542379201	NA	NA	0.946815284	0.542379201	NA	NA	NA	NA

comparison	k	prop	NMA		Direct		Indir		Diff		z	p
			TE	seTE	seTE	lower	TE	seTE	TE	seTE		
IM vsHSM	0	0	-0.47420104	0.417117737	NA	NA	-0.47420104	0.417117737	NA	NA	NA	NA
IM vsDDM	0	0	-0.441248232	0.364544141	NA	NA	-0.441248232	0.364544141	NA	NA	NA	NA
IM vsVM	0	0	-0.301811189	0.346529531	NA	NA	-0.301811189	0.346529531	NA	NA	NA	NA
IM vs MOX	0	0	-0.269334144	0.352407178	NA	NA	-0.269334144	0.352407178	NA	NA	NA	NA
IMvsWNM	0	0	-0.062378851	0.379230814	NA	NA	-0.062378851	0.379230814	NA	NA	NA	NA
IM vsCMT	0	0	-0.382675761	0.335095175	NA	NA	-0.382675761	0.335095175	NA	NA	NA	NA
IM vs CVT	1	1	0.14499687	0.306833277	0.14499687	0.306833277	NA	NA	NA	NA	NA	NA
IM vs PT	0	0	0.051111564	0.347173547	NA	NA	0.051111564	0.347173547	NA	NA	NA	NA
HSMvsDM	0	0	0.032952808	0.344363642	NA	NA	0.032952808	0.344363642	NA	NA	NA	NA
HSM vsVM	0	0	0.172389851	0.325233151	NA	NA	0.172389851	0.325233151	NA	NA	NA	NA
HSMvsMX	0	0	0.204866896	0.331488622	NA	NA	0.204866896	0.331488622	NA	NA	NA	NA
HSMvsWM	0	0	0.411822189	0.359874836	NA	NA	0.411822189	0.359874836	NA	NA	NA	NA
HSMvsCMT	0	0	0.091525279	0.313021825	NA	NA	0.091525279	0.313021825	NA	NA	NA	NA
HSMvsCVT	1	1	0.61919791	0.282560696	0.61919791	0.282560696	NA	NA	NA	NA	NA	NA
HSM vs PT	0	0	0.525312604	0.325919252	NA	NA	0.525312604	0.325919252	NA	NA	NA	NA
DDM vs VM	0	0	0.139437043	0.242276957	NA	NA	0.139437043	0.242276957	NA	NA	NA	NA
DDMvsMOX	0	0	0.171914088	0.250484197	NA	NA	0.171914088	0.250484197	NA	NA	NA	NA
DDMvsWNM	1	0.926147877	0.378869381	0.222798181	0.47301202	0.231510916	-0.801733183	0.819841976	1.274745203	0.851902677	1.496350743	0.134562278
DDM vs CMT	0	0	0.058572472	0.222367125	NA	NA	0.058572472	0.222367125	NA	NA	NA	NA
DDM vsCVT	1	0.709581686	0.586245102	0.196839455	0.77467784	0.233674302	0.125845695	0.36525835	0.648832145	0.433609665	1.496350743	0.134562278
DDM vs PT	1	0.45924036	0.492359797	0.216083255	0.14149756	0.318860769	0.790329705	0.293845456	-0.648832145	0.433609665	-1.496350743	0.134562278
HSM vs PT	0	0	0.525312604	0.325919252	NA	NA	0.525312604	0.325919252	NA	NA	NA	NA
DDM vsVM	0	0	0.139437043	0.242276957	NA	NA	0.139437043	0.242276957	NA	NA	NA	NA
VM vs MOX	0	0	0.032477045	0.224416424	NA	NA	0.032477045	0.224416424	NA	NA	NA	NA
VM vs WNM	0	0	0.239432338	0.26946468	NA	NA	0.239432338	0.26946468	NA	NA	NA	NA
VM vs CMT	0	0	-0.080864571	0.192786462	NA	NA	-0.080864571	0.192786462	NA	NA	NA	NA
VM vs CVT	2	0.756504517	0.446808059	0.16104675	0.463721635	0.185159587	0.394260078	0.326367205	0.069461557	0.375232761	0.185115918	0.853138117
VM vs PT	1	0.493252452	0.352922754	0.187599296	0.31772328	0.267113955	0.387184837	0.263533224	-0.069461557	0.375232761	-0.185115918	0.853138117
MOXvsWNM	0	0	0.206955293	0.2769248	NA	NA	0.206955293	0.2769248	NA	NA	NA	NA
MOX vs CMT	1	0.461744302	-0.113341616	0.186030915	0.0493877	0.27376905	-0.25293946	0.253565874	0.30232716	0.373155658	0.810190476	0.41783071
MOX vs CVT	1	0.60264965	0.414331014	0.173332511	0.34424596	0.223278843	0.520626965	0.274975011	-0.176381005	0.354209964	-0.49795608	0.618514999
MOX vs PT	1	0.514960363	0.320445709	0.197784561	0.31351918	0.275616674	0.327799515	0.283990475	-0.014280335	0.395746308	-0.036084569	0.971214926
WNM vsCMT	0	0	-0.320296909	0.253156436	NA	NA	-0.320296909	0.253156436	NA	NA	NA	NA
WNM vs CVT	1	0.925975357	0.207375721	0.222866217	0.30166582	0.231603184	-0.972100522	0.819136126	1.273766342	0.851248511	1.496350743	0.134562278
WNM vs PT	0	0	0.113490416	0.258508498	NA	NA	0.113490416	0.258508498	NA	NA	NA	NA

CMT vs CVT	3	0.69488064	0.527672631	0.134692673	0.461398645	0.16158047	0.678605398	0.243842325	-0.217206752	0.292518935	-0.742539119	0.45776076
CMT vs PT	2	0.731897887	0.433787325	0.152412225	0.536957475	0.178153581	0.152140851	0.294353825	0.384816624	0.344068122	1.118431492	0.263382751
CVT vs PT	0	0	0.093885306	0.162427867	NA	NA	0.093885306	0.162427867	NA	NA	NA	NA

comparison	k	prop	NMA		Direct		Indir		Diff		z	p
			TE	seTE	seTE	lower	TE	seTE	TE	seTE		
HSMvsDDM	0	0	-0.037028645	0.272611166	NA	NA	-0.037028645	0.272611166	NA	NA	NA	NA
HSM vs VM	0	0	0.069491442	0.31424457	NA	NA	0.069491442	0.31424457	NA	NA	NA	NA
HSM vs MOX	0	0	-0.054249682	0.363102897	NA	NA	-0.054249682	0.363102897	NA	NA	NA	NA
HSM vs WNM	0	0	0.12065528	0.2693596	NA	NA	0.12065528	0.2693596	NA	NA	NA	NA
HSM vs CMT	0	0	-0.005617534	0.267697698	NA	NA	-0.005617534	0.267697698	NA	NA	NA	NA
HSM vs CVT	2	1	0.280092878	0.205259108	0.280092878	0.205259108	NA	NA	NA	NA	NA	NA
DDM vs VM	1	0.575487546	0.106520087	0.239056699	0.3826418	0.315125069	-0.267802516	0.366906749	0.650444316	0.483657287	1.344845479	0.17867514
DDM vs MOX	0	0	-0.017221037	0.349138882	NA	NA	-0.017221037	0.349138882	NA	NA	NA	NA
DDM vsWNM	1	0.657839882	0.157683925	0.212069563	0.17778453	0.261467818	0.119038335	0.362546442	0.058746195	0.446995909	0.131424459	0.895439541
DDM vs CMT	0	0	0.03141111	0.248427662	NA	NA	0.03141111	0.248427662	NA	NA	NA	NA
DDM vs CVT	2	0.763397993	0.317121523	0.179403307	0.192200958	0.205331196	0.72017858	0.368825749	-0.527977622	0.422129521	-1.250747923	0.21102646
VM vs MOX	0	0	-0.123741124	0.382532038	NA	NA	-0.123741124	0.382532038	NA	NA	NA	NA
VM vs WNM	0	0	0.051163838	0.279944825	NA	NA	0.051163838	0.279944825	NA	NA	NA	NA
VM vs CMT	0	0	-0.075108977	0.293511677	NA	NA	-0.075108977	0.293511677	NA	NA	NA	NA
VM vs CVT	1	0.589235761	0.210601436	0.237946104	0.4777807	0.309980268	-0.172663616	0.371263525	0.650444316	0.483657287	1.344845479	0.17867514
MOX vsWNM	0	0	0.174904962	0.346605979	NA	NA	0.174904962	0.346605979	NA	NA	NA	NA
MOX vs CMT	0	0	0.048632147	0.345316042	NA	NA	0.048632147	0.345316042	NA	NA	NA	NA
MOX vs CVT	1	1	0.33434256	0.299520304	0.33434256	0.299520304	NA	NA	NA	NA	NA	NA
WNM vs CMT	0	0	-0.126272814	0.244855159	NA	NA	-0.126272814	0.244855159	NA	NA	NA	NA
WNM vs CVT	2	0.936744735	0.159437598	0.174422741	0.163108309	0.180215639	0.10507818	0.693513257	0.058030129	0.716546101	0.080985898	0.935453168
CMT vs CVT	3	1	0.285710413	0.171845153	0.285710413	0.171845153	NA	NA	NA	NA	NA	NA
